# Minutes of the Proceedings of the Committee, Appointed on the 14th of September, 1793, by the Citizens of Philadelphia, the Northern Liberties, and the District of Southwark, to Attend to and Alleviate the Sufferings of the Afflicted with the Malignant Fever, Prevalent in the City and Its Vicinity

**Published:** 1848-10

**Authors:** 


### BIBLIOGRAPHICAL NOTICES.

*Minutes of the Proceedings of the Committee, appointed on the 14th of September*, 1793, *by the Citizens of Philadelphia, the Northern Liberties, and the District of Southwark, to attend to and alleviate the Sufferings of the Afflicted with the Malignant Fever, prevalent in the City and its vicinity. With an Appendix.* Printed by order of the Select and Common Councils of the City of Philadelphia. 8vo. pp. 243. Philadelphia, 1848.

For this volume, of which a limited number only has been printed, we are indebted to a Committee of the Select and Common Councils of the City of Philadelphia, of which Charles A. Poulson, Esq.—himself a man of science—was the able and energetic chairman. The Committee were "appointed to inquire if there be any manuscripts relating to the city worthy of being printed, and report the same with the probable expense," and the result of their investigations was to select the "Minutes" before us, as having, in their opinion, "peculiar and paramount claims at this time to the attention of Councils." They, therefore, recommended, "that they be authorized to cause the said "Minutes" to be printed; and added—

"In relation to these 'Minutes,' your Committee deem it proper to state—that in addition to their intrinsic value as illustrations of the history of our city during a season of frightful calamity, and the almost total prostration of its government and industry—they furnish also appropriate means of gratitude—by perpetuating in a permanent form the records of the heroic self-devotion and chivalric deeds of those noble men who so fearlessly exposed themselves, amid disease and death, to rescue the lives and property of their fellow citizens from destruction, and for the care and protection of the helpless and pestilence-made orphans. Such testimony of gratitude is due, and especially to the memory of the venerated Stephen Girard; whose subsequent munificent benefactions, great as they are, have afforded no higher evidence of his benevolence, generosity and usefulness." p. 4.

Together with the daily records of the Committee of Citizens, the "Minutes" contain Reports by Doctors Physick, Leib, Cathrall, Annan, Duffield, Deveze, and others; a list of the names of the sick who were admitted, died, or were discharged at the hospital, Bush Hill; an account of the contributions in money, food, clothing and medicines for the use of the poor and sick received by the Committee from citizens of Philadelphia residing in the neighbourhood, and from the States of Pennsylvania, New Jersey, New York, Massachusetts, Delaware, Maryland, and Virginia; a list of the orphans, &c. &c.

We have looked over the "Minutes" with unmixed satisfaction, and are sure that the excellent Chairman of the Committee will pardon us when we say, that we should attempt in vain to depict the feelings, which the perusal has given rise to, in more just and eloquent language than in the following extract from the letter to the writer of this notice accompanying a presentation copy of the "Minutes:"

"The contents of the volume supply evidences, rarely found so eminently striking, of disinterested philanthropy striving against fearful and overwhelming obstacles, during the pestilence of 1793; unprecedented at least in the annals of our city.

"The beautiful examples exhibited in the conduct of the members of the 'Committee of Citizens,' should, I think, induce the most sceptical to correct an opinion, that the efforts of philanthropy are to be traced to no other motives than those ascribed to the hope of reward, or the fear of punishment, *two* incentives to virtuous deeds, it is true, too much relied on, perhaps because they are inculcated with our earliest perceptions in the nursery, and cease not to sound in our ears even from 'high places' to the close of our probation.

"The conduct of the noble men, the 'Minutes' of whose proceedings you have in this volume, presents to my mind a moral grandeur vainly sought for in the battle field, or elsewhere, where glory is said to be found; indeed, nowhere so pure, as where 'Stephen Girard, and Peter Helm at the hospital,' and their colleagues were found, for successive weeks, like 'ministering angels' doing good to the destitute and sick! The first and greatest of all admonitions could alone have impelled and sustained them, under such discouraging and terrific circmstances."

